# High Concentrations of Polyelectrolyte Complex Nanoparticles Decrease Activity of Osteoclasts

**DOI:** 10.3390/molecules24122346

**Published:** 2019-06-25

**Authors:** Vivien Kauschke, Felix Maximilian Hessland, David Vehlow, Martin Müller, Christian Heiss, Katrin Susanne Lips

**Affiliations:** 1 Experimental Trauma Surgery, Justus-Liebig-University Giessen, Aulweg 128, 35392 Giessen, Germany; Vivien.Kauschke@chiru.med.uni-giessen.de (V.K.); felix.hessland@t-online.de (F.M.); Christian.Heiss@chiru.med.uni-giessen.de (C.H.); 2 Institute and Outpatient Clinic for Occupational and Social Medicine, University Hospital of Giessen-Marburg GmbH, Campus: Giessen, Aulweg 129, 35392 Giessen, Germany; 3 Leibniz Institute of Polymer Research Dresden, Hohe Strasse 6, 01069 Dresden, Germany; Vehlow@ipfdd.de (D.V.); mamuller@ipfdd.de (M.M.); 4 Department of Trauma, Hand and Reconstructive Surgery, University Hospital of Giessen-Marburg GmbH, Campus: Giessen, Rudolf-Buchheim-Strasse 7, 35392 Giessen, Germany

**Keywords:** osteoclasts, polyelectrolyte complex nanoparticles, BDNF, drug delivery system, cathepsin K, calcitonin receptor, tartrate-resistant acidic phosphatase 5b, pit formation

## Abstract

Fracture treatment in osteoporotic patients is still challenging. Osteoporosis emerges when there is an imbalance between bone formation and resorption in favor of resorption by osteoclasts. Thus, new implant materials for osteoporotic fracture treatment should promote bone formation and reduce bone resorption. Nanoparticles can serve as drug delivery systems for growth factors like Brain-Derived Neurotrophic Factor (BDNF), which stimulated osteoblast differentiation. Therefore, polyelectrolyte complex nanoparticles (PEC-NPs) consisting of poly(l-lysine) (PLL) and cellulose sulfate (CS), with or without addition of BDNF, were used to analyze their effect on osteoclasts in vitro. Live cell images showed that osteoclast numbers decreased after application of high PLL/CS PEC-NPs concentrations independent of whether BDNF was added or not. Real-time RT-PCR revealed that relative mRNA expression of cathepsin K and calcitonin receptor significantly declined after incubation of osteoclasts with high concentrations of PLL/CS PEC-NPs. Furthermore, Enzyme-Linked Immunosorbent Assay indicated that tartrate-resistant acidic phosphatase 5b activity was significantly reduced in the presence of high PLL/CS PEC-NPs concentrations. Consistent with these results, the pit formation analysis showed that less hydroxyapatite was resorbed by osteoclasts after incubation with high concentrations of PLL/CS PEC-NPs. BDNF had no influence on osteoclasts. We conclude that highly concentrated PLL/CS PEC-NPs dosages decreased osteoclastogenesis and osteoclasts activity. Moreover, BDNF might be a promising growth factor for osteoporotic fracture treatment since it did not increase osteoclast activity.

## 1. Introduction

Osteoclasts are an important cell type involved in bone metabolism. As an opponent to osteoblasts, they resorb bone in order to repair small defects and are involved in the remodeling process of aged parts of the bone matrix [1,2]. Bone formation is accomplished by osteoblasts [3]. During the bone formation process, osteoblasts immure themselves into the bone matrix where they differentiate into osteocytes [3]. Osteocytes are responsible for sensing the mechanical load and regulating bone modeling according to the mechanical needs of bone [4].

In osteoporosis, there is an imbalance between bone formation and bone resorption in favor of resorption by osteoclasts. This imbalance causes reduced bone mineral density, pathological alterations of bone microarchitecture and a subsequent increased fracture risk [5]. Osteoblasts differentiate from mesenchymal stem cells (MSCs). In patients with osteoporosis, the ability of MSCs to differentiate into osteoblasts is reduced since they predominantly differentiate into adipocytes [6]. This causes a decrease in bone formation and delayed fracture healing [7,8]. Epidemiological studies revealed an increasing risk of osteoporotic fractures in the future [9,10].

The treatment of osteoporotic fractures is challenging since properties of osteoporotic bone facilitate loosening of implant materials [11]. Stable fixation is required to allow bone formation by osteoblasts. Bridging of the fracture gap with an osteoinductive bone substitute material supports fracture healing [12]. Therefore, the demand for osteoinductive bone substitute materials increased [13]. Osteoinductivity of bone substitute materials can be improved by adding growth factors that stimulate bone formation [14]. The development of bone substitute materials functionalized with growth factors holds new challenges in terms of a controlled continuous release over a defined period of time, in a certain concentration and a rate adjusted to conditions required for osteogenesis [15]. Moreover, bioactivity of the growth factor should be preserved during production, storage and implantation of the carrier material into the fracture gap [16]. Several nanoparticles were described as suitable drug delivery systems [17]. Here, we focused on polyelectrolyte complex nanoparticles (PEC-NPs) that consist of poly(l-lysine) (PLL) and cellulose sulfate (CS) [18]. Vehlow et al. confirmed biocompatibility of PLL/CS PEC-NPs with human mesenchymal stem cells (hMSCs) in vitro [18]. Thus, the aim of the present study was to analyze the effect of PLL/CS PEC-NPs on osteoclasts.

Osteoclasts are of hematopoietic origin and derive from monocytes [2]. Monocytes can differentiate into osteoclasts after stimulation with macrophage colony stimulation factor (M-CSF) and Receptor Activator of Nuclear Factor Kappa B Ligand (RANKL) [19]. Osteoclasts can be detected via tartrate-resistant acidic phosphatase (TRAP) 5b, cathepsin K or calcitonin receptor expression. TRAP5b is one of the prominent enzymes involved in bone resorption [20]. It is active at the ruffled border of osteoclasts and often associated with the enzyme cathepsin K which cleaves type I collagen of bone [20]. The calcitonin receptor (CALCR) is localized on the plasma membrane of osteoclasts where it inhibits bone resorption by binding calcitonin [21]. This receptor is present on mature osteoclasts and absent on osteoclast precursors such as monocytes and macrophages [22], which makes CALCR a good osteoclast marker.

Besides verification of nanoparticles biocompatibility, it is necessary to analyze the impact of the substance that should later be delivered by the nanoparticles. PEC-NPs can release drugs in a controlled manner with regard to time and concentration.

Because of the imbalance in favor of osteoclastic bone resorption, treatment of osteoporotic patients aims at inhibiting osteoclasts. There are therapies addressing this issue for example through administration of bisphosphonates, calcitonin or anti-RANKL antibodies [23,24,25]. Although these treatments are helpful, there is still a need for new agents due to side effects of currently available therapies. We decided to focus on Brain-Derived Neurotrophic Factor (BDNF), which is a well-known growth factor in the nervous system, where it regulates outgrowth and survival of neurons and angiogenesis [26]. However, Yamashiro et al. showed that BDNF mRNA and protein were present in osteoblasts [27]. Moreover, Asaumi et al. and Kilian et al. measured an increase of BDNF during murine [28] and human fracture healing [29], respectively. Thus, BDNF seems to be involved in fracture healing. In addition, BDNF significantly increased osteogenic differentiation of hMSCs in vitro and new bone formation in vivo [30,31]. Therefore, BDNF appears to be a suitable growth factor to be incorporated into bone substitute materials and drug delivery systems. However, if BDNF stimulates bone degradation by osteoclasts as well, the positive anabolic effect on bone formation by osteoblasts would be neutralized. In patients with multiple myeloma, BDNF was identified as a factor that contributed to osteolytic bone destructions and increased osteoclast formation [32,33]. This effect was blocked in vitro by inhibiting the highly BDNF-specific receptor tyrosine receptor kinase B (TrkB) using K252a [33] or specific small interfering RNA for TrkB [32]. Nevertheless, in contrast to patients with multiple myeloma, in peripheral blood cells of healthy young donors, BDNF application did not increase osteoclast formation in vitro [34]. Because of these reports, it is necessary to analyze in more detail the effect of BDNF on osteoclasts and its suitability in combination with drug delivery systems. Thus, we investigated here the effect of BDNF and PLL/CS PEC-NPs on osteoclasts in vitro. This is an important first step for future analyses of BDNF loaded PLL/CS PEC-NPs that might be used for functionalization of paste-like calcium phosphate bone cements that are clinically applied for the augmentation of osteoporotic fractures.

## 2. Results

### 2.1. Live Cell Imaging of Osteoclastogenesis

**Figure 1:**
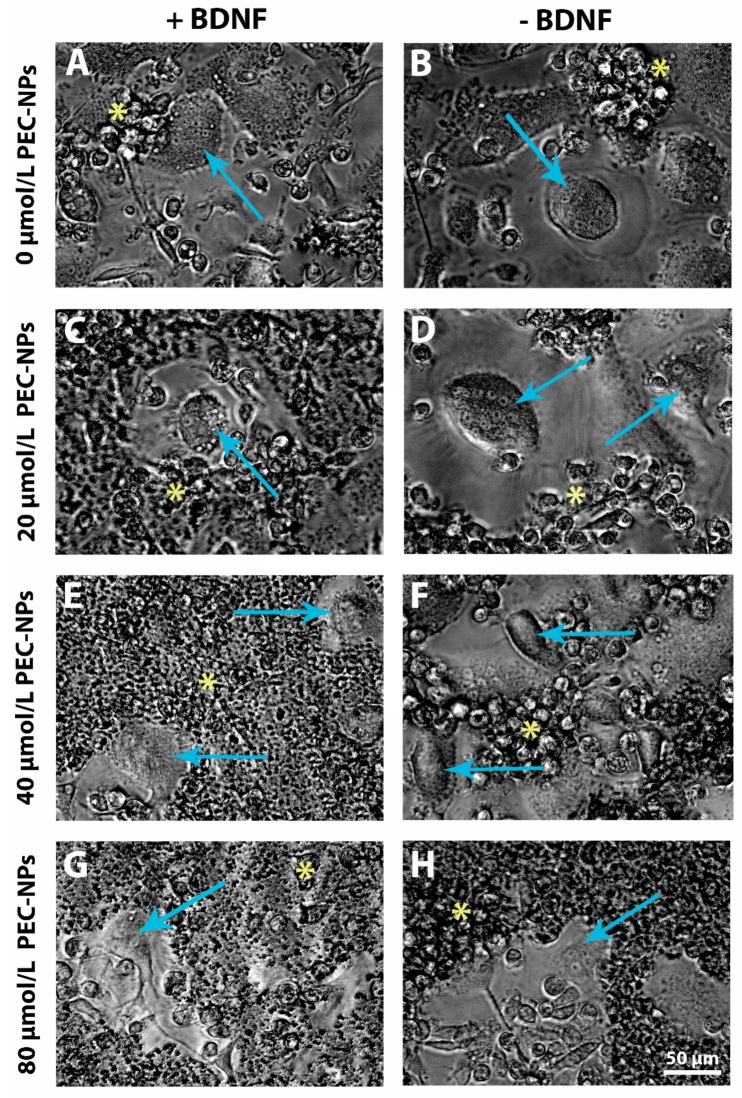
Live cell images of osteoclastogenesis: osteoclasts and monocytes were depicted after incubation with no PLL/CS PEC-NPs in the presence of 40 ng/mL BDNF (**A**) or in absence of BDNF (**B**) and after incubation with 20, 40 or 80 µmol/L PLL/CS PEC-NPs either with BDNF (**C**, **E**, **G**) or without BDNF (**D**, **F** and **H**). Blue arrows indicate osteoclasts and yellow asterisks monocytes. The scale bar shown in H applies to all images in the figure.

Differentiation of monocytes into osteoclasts was analyzed daily using a light microscope. After seven days of incubation in differentiation medium without addition of PLL/CS PEC-NPs, we observed multinucleated cells with 5–10 nuclei. Cell size was approximately 20–50 µm in diameter. Several monocytes were still present in cultures. No differences were visible between cells that were incubated with or without BDNF (Figure 1A,B). After addition of 20 µmol/L PLL/CS PEC-NPs with or without BDNF, the appearance of multinucleated cells with a size of 20–50 µm and having 5–10 nuclei was not abrogated (Figure 1C,D). Nevertheless, the number of multinucleated cells seemed less and the fraction of monocytes more compared to non-treated controls shown in Figure 1A,B. After addition of BDNF to 20 µmol/L PLL/CS PEC-NPs, multinucleated cells appeared smaller and the number of monocytes comparatively higher (Figure 1C). In the presence of 40 µmol/L PLL/CS PEC-NPs and BDNF, the number of multinucleated cells further decreased and the number of monocytes was proportionally higher (Figure 1E). Application of 40 µmol/L PLL/CS PEC-NPs without BDNF resulted in smaller multinucleated cells with less nuclei, ranging from 4–8 nuclei per cell and a higher amount of monocytes (Figure 1F). In line with these observations, the number of multinucleated cells further decreased and the portion of monocytes increased after application of 80 µmol/L PLL/CS PEC-NPs independent of whether BDNF was added to cultures or not (Figure 1G,H).

### 2.2. Detection of Cathepsin K and Calcitonin Receptor mRNA by Real-Time Reverse Transcriptase (RT)-Polymerase Chain Reaction (PCR)

**Figure 2:**
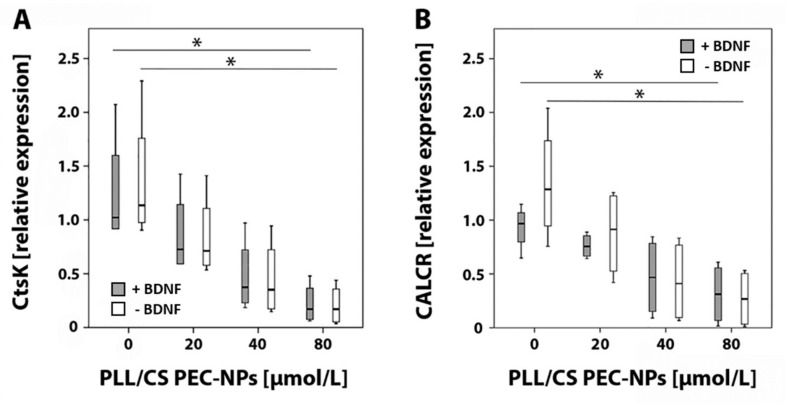
Real-time RT-PCR: (**A**) relative mRNA expression of cathepsin K (CtsK) and (**B**) calcitonin receptor (CALCR) in osteoclasts after seven days of in vitro culture with increasing PLL/CS PEC-NPs concentrations (0, 20, 40 and 80 µmol/L). The asterisk (*) indicates statistically significant differences with a likelihood of *p* ≤ 0.05.

After seven days of cultivation, cells of four different donors were harvested from osteo assay plates and mRNA specific for the osteoclast markers cathepsin K (CtsK) and calcitonin receptor (CALCR) were analyzed by real-time RT-PCR. A graded decrease in relative expression of CtsK and CALCR was detected with increasing PLL/CS PEC-NPs concentrations compared to controls treated without PLL/CS PEC-NPs. Relative mRNA expression of CtsK and CALCR was significantly reduced when cells were exposed to 80 µmol/L PLL/CS PEC-NPs either with or without addition of BDNF compared to controls that received no PLL/CS PEC-NPs (Figure 2).

### 2.3. Detection of Osteoclast Specific Tartrate-Resistant Acidic Phosphatase (TRAP) Isoform 5b

**Figure 3:**
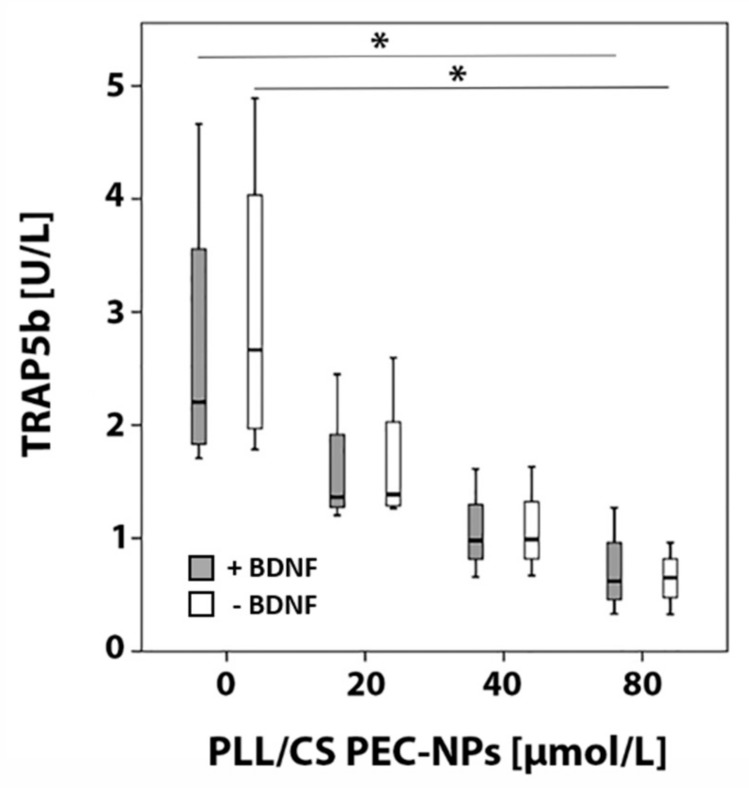
TRAP5b ELISA: detection of TRAP5b activity in osteoclasts after seven days of in vitro culture with different concentrations of PLL/CS PEC-NPs (0, 20, 40 and 80 µmol/L) with or without BDNF. The asterisk (*) indicates statistically significant differences with a likelihood of *p* ≤ 0.05.

TRAP5b was detected in cell culture medium of four different donors after seven days using an Enzyme-Linked Immunosorbent Assay (ELISA). With increasing PLL/CS PEC-NPs concentration, a graded decrease of TRAP5b activity was measured compared to controls that were incubated without PLL/CS PEC-NPs. A significant decrease of TRAP5b activity was detected when cells were exposed to 80 µmol/L PLL/CS PEC-NPs either with or without addition of BDNF compared to respective controls that were treated without PLL/CS PEC-NPs (Figure 3).

### 2.4. Pit Formation Assay

**Figure 4:**
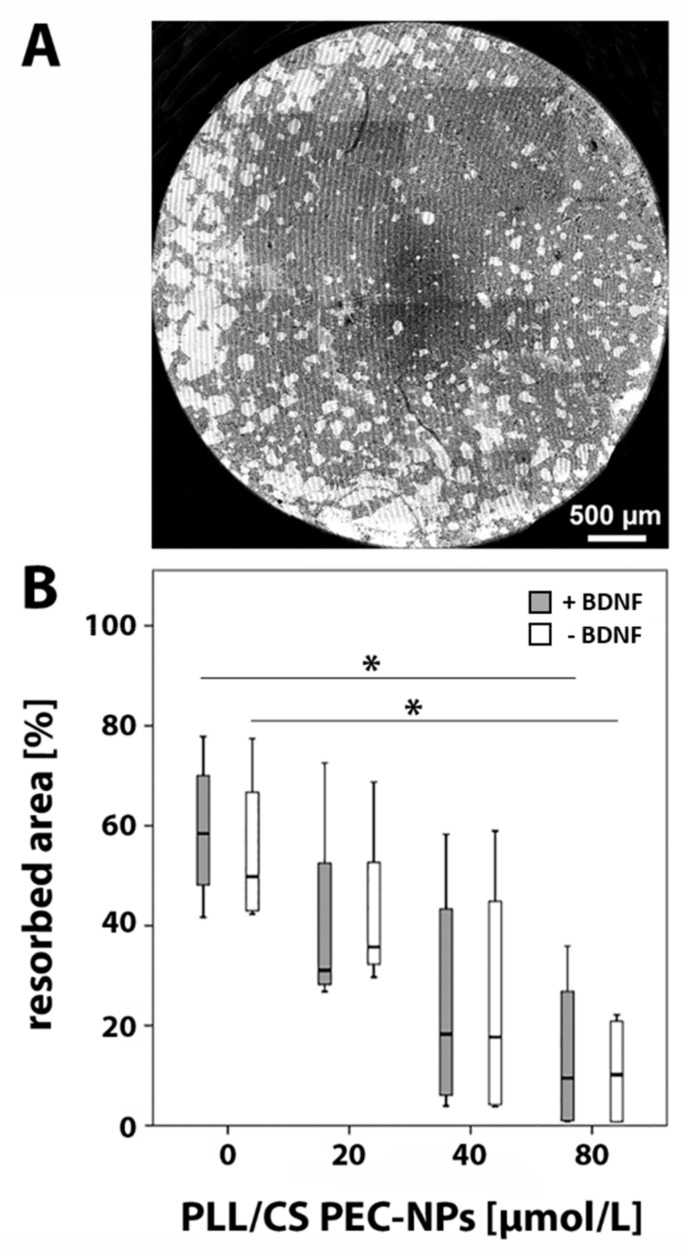
Pit formation assay: (**A**) overview image of a well with hydroxyapatite coating after resorption by osteoclasts; resorbed parts are shown in white; (**B**) resorbed area in percent after seven days of in vitro culture of osteoclasts with 0, 20, 40 and 80 µmol/L PLL/CS PEC-NPs with or without BDNF. The asterisk (*) indicates statistically significant differences with a likelihood of *p* ≤ 0.05.

Resorption lacunae produced by osteoclasts on osteo assay plates were quantified. A graded decrease of the resorbed area was measured with increasing concentrations of PLL/CS PEC-NPs compared to controls that were not subjected to PLL/CS PEC-NPs. Significantly less hydroxyapatite was resorbed when cells were treated with 80 µmol/L PLL/CS PEC-NPs compared to controls incubated without PLL/CS PEC-NPs with or without BDNF (Figure 4).

## 3. Discussion

Aim of this study was to analyze the compatibility of PLL/CS PEC-NPs and additionally administered BDNF on osteoclasts in vitro. The analysis was based on live cell imaging, real-time RT-PCR as well as a TRAP5b ELISA and pit formation assay.

Using light microscopy, multinucleated cells were observed in cell cultures indicating osteoclasts. To verify this observation, real-time RT-PCR was performed to quantify the mRNA amount of CtsK and CALCR, which are specific osteoclast markers [35]. Monocytes were differentiated into osteoclasts by adding RANKL and M-CSF to the cell culture medium for seven days. Several studies suggested longer culturing times for osteoclast differentiation [36,37,38]. However, in an in vitro pilot test, we harvested osteoclasts daily over a period of 12 days (*n* = 1) and found that, at day 12, a high amount of hydroxyapatite was already degraded. We hypothesized that osteoclast activity might increase after addition of PLL/CS PEC-NPs and BDNF as shown by Brulefert et al. for polyethylene nanoparticles [39]. Thus, changes between the groups might have not been measureable since in controls a high amount of the hydroxyapatite had been resorbed already. Therefore, we decided to harvest osteoclasts after seven days. The Human Monocyte Enrichment Kit used for the isolation of monocytes equipped with Magnetic Particles that removed all non-monocytic cells from the suspension might be the reason for the short differentiation time of osteoclasts in our study. Starting the differentiation with a more pure cell suspension seems to accelerate the differentiation.

Observations of live cell images revealed phenotypic differences of multinucleated cells with increasing PLL/CS PEC-NPs concentration. In addition, the number of monocytes appeared to be higher than the number of multinucleated cells, indicating inhibition of osteoclastogensis.

Our real-time RT-PCR results showed that relative mRNA expression of CtsK and CALCR was significantly decreased after seven days of incubation with 80 µmol/L PLL/CS PEC-NPs with or without BDNF. CtsK is a member of the cysteine protease family and predominantly found in lysosomes of osteoclasts. It is able to degrade type I collagen in an acidic environment [40]. Lately, inhibition of CtsK has attracted general interest because it is an effective antiresorptive treatment that does not interfere with bone formation unlike other bone resorption inhibiting drugs [41]. In this study, we measured a graded decrease in relative CtsK expression after application of increasing PLL/CS PEC-NPs concentrations.

The calcitonin receptor is expressed on osteoclasts and, via binding of calcitonin, it inhibits bone resorption [42]. This is accomplished by calcitonin inducing loss of the ruffled border [43,44], preventing osteoclast formation [45] and subsequently declining osteoclast numbers [42]. However, calcitonin administration for osteoporosis treatment is not recommended because of limited effectiveness in fracture healing and its association with cancer risk [46]. Here, we showed that relative mRNA expression of the calcitonin receptor significantly decreased after application of 80 µmol/L of PLL/CS PEC-NPs. This indicates either that the number of osteoclasts might have been significantly reduced or that monocytes did not differentiate into osteoclasts as already estimated during live cell imaging.

By analyzing TRAP5b, it is possible to determine the osteoclast activity. TRAP5b is another enzyme that is specific for osteoclasts. It is located in the ruffled border and resorptive space where it is involved in the bone resorption process [20]. Using an ELISA, we showed that TRAP5b activity significantly declined after application of a high PLL/CS PEC-NPs concentration suggesting that either osteoclasts were less active or less osteoclasts were present.

Moreover, we conducted a pit formation assay to verify activity via resorption of hydroxyapatite by osteoclasts. Results of the pit formation assay were compliant with results of the TRAP5b ELISA as well as real-time RT-PCR. It showed that significantly less hydroxyapatite was resorbed with increasing PLL/CS PEC-NPs concentration.

In the present study, high concentrations of PLL/CS PEC-NPs inhibited osteoclasts activity. Nanoparticles were described as being promising drug delivery systems [47]. One requirement of a suitable drug delivery system is its biocompatibility with cells or tissue of interest. Since PEC-NPs can serve as drug delivery systems, loading with growth factors could additionally stimulate bone formation. The benefit of using nanoparticles as drug delivery systems is the possibility to adjust the concentration, time point and duration of growth factor release at the fracture side. Several drugs are known that enhance bone formation, e.g., bone morphogenic protein 2 (BMP2). However, BMP2 also induced inflammation [48]. Thus, there still is a demand for new growth factors with no risk of causing side effects. The growth factor BDNF, which is involved in the development of the nervous system, also promoted osteoblast differentiation in vitro [30]. As shown in another study of our group, BDNF significantly increased differentiation of hMSCs into osteoblasts after addition of 40 ng/mL BDNF into cell culture medium [30]. Moreover, new bone formation significantly increased during murine fracture healing after functionalizing a calcium phosphate cement with BDNF as shown previously by our group [31]. Since 40 ng/mL BDNF had a significant effect on osteoblasts, we decided to use this concentration for experiments of the current study with osteoclasts. These experiments revealed that additional administration of BDNF did not stimulate osteoclasts activity, which would reverse the inhibitory effect of PLL/CS PEC-NPs. This suggests that BDNF might be a suitable growth factor for the functionalization of PEC-NPs that can be incorporated into implant materials for osteoporotic fracture treatment.

Functionalization of implant materials with growth factors or drugs has the advantage of reduced side effects compared to systemic administration and that growth factors have direct contact to cells of interest. Growth factors either can be attached to the surface of an implant material or be mixed into the fluid phase before the biomaterial sets or be bound to nanoparticles [16]. However, the release and remaining activity of the growth factor are still improvable. Currently, large amounts of growth factors are utilized due to fast inactivation of growth factors. Moreover, the growth factor release by the carrier material is not optimized yet, which should comply with requirements of the cell of interest [16]. Thus, the quest for suitable drug delivery systems increased.

Summarizing all results of this study, a high PLL/CS PEC-NPs concentration changed the phenotypic appearance of osteoclasts and significantly decreased the expression of specific osteoclast markers, indicating inhibition of osteoclasts. Additional application of BDNF did not alter this result.

## 4. Materials and Methods

To analyze osteoclasts activity, live cell imaging, real-time RT-PCR, a TRAP5b ELISA and pit formation assay were performed.

Cathepsin K cleaves collagen fibers of bone matrix and was addressed in the present study by real-time RT-PCR. On mRNA level, we also determined the expression of calcitonin receptor that is localized on the plasma membrane of osteoclasts and therefore a good marker for the presence of osteoclasts. TRAP5b is an osteoclast-specific enzyme, which indicates osteoclast activity. Moreover, osteoclast activity can be analyzed by measuring the resorbed area using a pit formation assay.

### 4.1. Polyelectrolyte Complex Nanoparticles

Polyelectrolyte Complex Nanoparticles (PEC-NPs) consisted of the cationic (*n*+) polypeptide poly(l)-lysine (PLL, 30,000–70,000 g/mol, Sigma Aldrich, St. Louis, MO, USA) and anionic (*n*-) cellulose sulfate (CS) with an average degree of substitution (DS) of 1.0 further denoted as CS-1.0. In detail, we used a mixture of high (CS-3.0, 1,200,000 g/mol, Janssen Chimica, Beerse, Belgium) and low (CS-0.5, without declaration of molecular weight, Euroferm, Erlangen, Germany) substituted cellulose. PLL/CS PEC-NPs were prepared by mixing 0.002 M PLL and CS-1.0 solutions resulting in a molar mixing ratio of *n*−/*n*+ = 1.1. The equation for calculating the stoichiometric mixing ratio defining *n*-/*n*+ was recently given by Vehlow et al. [18]. The pH was kept constant at a certain value by adding NaOH or HCl solution. PLL/CS PEC-NPs size ranged from 100 to 200 nm. For the experiments, the PLL/CS solution was diluted in cell culture medium to concentrations of 20, 40 and 80 µmol/L PLL/CS PEC-NPs.

### 4.2. In Vitro Culture of Osteoclasts

Osteoclasts were obtained after differentiation of monocytes isolated from Peripheral Blood Mononuclear Cells (PBMCs) of blood samples from four different donors (*n* = 4), which were provided by the Institute of Clinical Immunology and Transfusion Medicine at the Justus-Liebig-University Giessen (Giessen, Germany). Permission to use donor blood was granted by a written statement of the local ethics commission of the medical faculty of the Justus-Liebig-University Giessen, Germany (05/00). In detail, 20 mL blood were diluted in 15 mL cold phosphate buffered saline (PBS, Gibco, Life Technologies, Carlsbad, CA, USA) including 2% fetal bovine calf serum (FBS, Biochrom, Berlin, Germany) and 1 mM ethylenediaminetetraacetic acid (EDTA, Roth, Karlsruhe, Germany). Subsequently, samples were transferred into a Leucosep centrifuge tube (Greiner Bio-One, Frickenhausen, Germany) and centrifuged for 15 min at 800× *g* without activating the break. Supernatant was removed, PBMCs were obtained, washed with 30 mL cold PBS and centrifuged for 5 min at 450× *g* before cold PBS was added and PBMCs washed and centrifuged again for 10 min at 120× *g*. Finally, PBMCs were washed and centrifuged a further time for 10 min at 120× *g* before they were resuspended in 500 µL cold PBS. Cell number was determined using a cell counting machine (Sysmex, KX-21N, Norderstedt, Germany). PBMCs were then diluted in PBS to a concentration of 5 × 10^7^ cells/mL. To isolate monocytes from PBMCs, 50 µL of the EasySep™ Human Monocyte Enrichment Kit (Stemcell Technologies, Vancouver, Canada) were added to PBMCs, shaken and incubated for 10 min at 2–8 °C. Following this, 50 µL of EasySep™ Magnetic Particles (Stemcell Technologies) were admixed and incubated for 5 min at 2–8 °C. Then, 2.5 mL PBS were added, mixed and placed in an EasySep™ magnet (Stemcell) for 2.5 min at room temperature. Afterwards, monocytes were transferred into a new Leucosep centrifuge tube (Greiner Bio-One) and centrifuged for 5 min at 450× *g*. Supernatant was decanted, 1 mL PBS added and a 1:10 cell dilution made for counting monocytes using the Sysmex cell counting machine. Finally, 100,000 monocytes were seeded onto Corning^®^ osteo assay surface 96-well plates (Corning incorporated, Corning, NY, USA) filled with 200 µL medium. Medium consisted of alpha modified minimal essential medium (αMEM, Gibco) supplemented with 10% FBS (Biochrom), 1% of 100 U/mL penicillin and 100 µg/g streptomycin (Gibco) as well as 50 ng/mL RANKL (Santa Cruz Biotechnology Inc., Dallas, TX, USA) and 50 ng/mL M-CSF (Sigma-Aldrich, St. Louis, MO, USA). Monocytes were incubated with 0, 20, 40 or 80 µmol/L PLL/CS PEC-NPs with or without addition of 40 ng/mL BDNF into cell culture medium (PAN Biotech, Aidenbach, Germany) at 37 °C and 5% CO_2_ for seven days.

### 4.3. Live Cell Imaging

Cells were observed with an inverse light microscope (Axiovert 10, Zeiss, Oberkochen, Germany) and photographed using the associated Stingray F-145 camera (Allied Vision Technologies GmbH, Stadtroda, Germany).

### 4.4. Real-Time Reverse Transcriptase (RT)-Polymerase Chain Reaction (PCR)

RNA was isolated using the RNeasy Mini Kit (Qiagen, Hilden, Germany) according to the manufacturer’s protocol. For mRNA anaylsis, 500 ng of RNA were reverse transcribed into cDNA using the Quantitect Reverse Transcription Kit (Qiagen, Hilden, Germany) and a cycler (TC-3000, Techne, Bibby Scientific, Burlington, NJ, USA). The mRNA expression of cathepsin K (CtsK) and calcitonin receptor (CALCR) was analyzed by real-time RT-PCR using the Roche LightCycler^®^ FastStart DNA Master^Plus^ SYBR Green I (Roche, Basel, Switzerland) and a LightCycler^®^ 2.0 instrument (Roche). Primers used are listed in Table 1. The cycling procedure started with heating for 10 min at 95 °C, followed by 40 cycles of denaturation for 5 s at 95 °C, annealing for 5 s at 60 °C and elongation for 5 s at 72 °C. Purity of the RT-PCR products was verified by a melting curve. Samples that were not reverse transcribed or received water instead of cDNA served as negative controls. Results were evaluated using delta threshold cycle (CT) values and referenced to the housekeeping gene beta-2-microglobulin (B2M).

### 4.5. Tartrate-Resistant Acidic Phosphatase (TRAP) 5b Enzyme-Linked Immunosorbent Assay (ELISA)

TRAP5b is one of the prominent enzymes that is synthesized by osteoclasts and involved in degradation of bone matrix.

To determine the osteoclast specific isoform TRAP5b in sample supernatants, the Microvue TRAP5b ELISA Kit (Quidel, San Diego, CA, USA) was used according to the manufacturer’s manual. In brief, 50 µL of isoform dilution buffer (kit component) was added to 50 µL of cell culture medium and mixed for 60 min at 300 U/min and room temperature. Subsequently, wells were washed 3× using wash buffer (kit component) before cells were incubated with 100 µL substrate for 60 min at 37 °C. Finally, the reaction was stopped by adding 50 µL stop solution (kit component) and measured using a plate reader (BioTek, Bad Friedrichshall, Germany) at 405 nm.

### 4.6. Pit Formation Assay

To analyze the activity of osteoclasts, a pit formation assay was conducted in which osteoclasts resorb a thin layer of hydroxyapatite leaving resorption pits, which were measured histomorphometrically.

Therefore, 100,000 monocytes were seeded on 96-well Corning^®^ osteo assay surface well plates (Corning), incubated in osteoclast incubation medium as described above (4.2) and cultured for seven days. Subsequently, wells were washed with aqua dest, incubated for 5 min with 150 µL of 1.25% sodium-hypochlorite, washed again with aqua dest and dried overnight. Since the bottom of the osteo assay surface plates were covered with calcium phosphate, which osteoclasts resorbed, the resorption pits were visible. Wells were analyzed with 40× magnification and phase contrast of an inverse light microscope (Zeiss) and pictures taken with the appurtenant Stingray F-145 camera (Allied Vision Technologies GmbH). Single pictures were combined to one image and resorption pits measured using Adobe Photoshop (CS6, Adobe Systems Incorporated, San Jose, CA, USA). The total well was defined as region of interest (ROI) and the resorbed surface was referenced to the total ROI and calculated as a percentage.

### 4.7. Statistical Analysis

Statistical analysis and generation of graphs were carried out using the statistics program SPSS (version 23.0, SPSS Institute Inc., Chicago, IL, USA). Results of the real-time RT-PCR analysis, TRAP5b ELISA and pit formation assay were analyzed with Kolmogorov–Smirnov-, Kruskal–Wallis- and Friedman tests. A value of *p* ≤ 0.05 was considered as significant. Sample size (*n*) was four.

## 5. Conclusions

We conclude that a high PLL/CS PEC-NPs concentration declined either osteoclast activity or the differentiation of monocytes into osteoclasts. Considering previous studies where BDNF promoted osteoblast differentiation, BDNF appears to be a promising growth factor for application in osteoporosis treatment since it did not stimulate osteoclasts. Further in vivo testing is necessary to verify this result and analyze the suitability of PLL/CS PEC-NPs and BDNF for the incorporation into bone substitute materials to treat osteoporotic fractures.

## Figures and Tables

**Table 1:** Human primers used for real-time RT-PCR analysis.

Primer	Sequence	Length [bp]	Accession No.
Cathepsin K	for GCG ATA ATC TGA ACC ATG CG rev TTG TTT CCC CAG TTT TCT CCC	103	NM_000396.3
Calcitonin Receptor	for TGA GTG TGG AAA CCC ATT TGC rev ATT TTG GTC ACA AGC ACC CG	109	NM_001164737.1
Beta-2-microglubulin	for TCT CTC TTT CTG GCC TGG AG rev CAA CTT CAA TGT CGG ATG GA	135	NM_004048.2

for: forward primer; rev: reverse primer.
